# Discriminating Tonate Virus from Dengue Virus Infection: A Matched Case–Control Study in French Guiana, 2003–2016

**DOI:** 10.4269/ajtmh.19-0156

**Published:** 2019-11-25

**Authors:** Rémi Mutricy, Félix Djossou, Séverine Matheus, Enguerrane Lorenzi-Martinez, Franck De Laval, Magalie Demar, Mathieu Nacher, Dominique Rousset, Loïc Epelboin

**Affiliations:** 1 Unité des Maladies Infectieuses et Tropicales, Centre Hospitalier Andrée Rosemon, Cayenne, French Guiana;; 2 Equipe EA 3593, Ecosystèmes Amazoniens et Pathologie Tropicale, Université de la Guyane, Cayenne, French Guiana;; 3 Centre National de Référence des Arbovirus, Institut Pasteur de la Guyane, Cayenne, French Guiana;; 4 Centre Médical Interarmées (CMIA), Cayenne, French Guiana;; 5 Laboratoire Hospitalo-Universitaire de Parasitologie et Mycologie, Centre Hospitalier Andrée Rosemon, Cayenne, French Guiana;; 6 Centre D’Investigation Clinique (CIC INSERM 1424), Centre Hospitalier Andrée Rosemon, Cayenne, French Guiana

## Abstract

Tonate virus (TONV) is an arbovirus discovered in 1973 in French Guiana (FG) belonging to the Venezuelan equine encephalitis virus complex, Alphavirus genus. Only few publications and cases have been reported in FG. The objectives of the present study were to describe the clinical picture of TONV and to compare its presentation with that of dengue virus (DENV). A retrospective study was performed in Cayenne hospital from 2003 to 2016 including all patients exclusively positive for TONV IgM and not for other alphaviruses. They were classified as high probability: typical clinical picture of arbovirus infection (i.e., fever, chills, headaches, muscle, and joint pains) and IgM seroconversion; medium probability: typical clinical picture + single positive IgM on a unique serum sample without control; and low probability: atypical clinical picture of infection and single positive IgM. Only patients with high and medium probability were included in the analysis and compared with a gender- and age-matched control group of DENV diagnosed by NS1 antigen (two controls per case). During the study period, 45 cases of TONV were included and compared with 90 cases of DENV. Twenty-eight (62.2%) were men; the median age was 34 years (IQ [22–49]). In the bivariate analysis, variables significantly associated with TONV versus DENV were the presence of cough (33.3% versus 10.3%) and anemia (32.5% versus 11.1%) and the absence of nausea (4.4% versus 32.2%), rash (2.2% versus 27.4%), fatigue (17.8% versus 41.0%), anorexia (6.7% versus 30.1%), muscle pain (42.2% versus 61.4%), headache (53.3% versus 70.8%), leukopenia (9.8% versus 44.4), and lymphopenia (42.5% versus 89.9%). There were no cases with severe neurological involvement, and there were no deaths. Tonate virus may be evoked as a cause of fever in patients living or returning from the Amazonian area. Positive TONV IgM does not prove the diagnosis and should not preclude from searching for alternative infectious diagnoses.

## INTRODUCTION

The Tonate virus (TONV) is an arthropod-borne virus of the *Togaviridae* family and the Alphavirus genus. It belongs to the subtype IIIB of the Venezuelan equine encephalitis (VEE) complex viruses.^1^ It was isolated for the first time in January 1973 from a bird, the crested oropendola (*Psarocolius decumanus*), in Tonate, a village located 30 km northwest of Cayenne, the capital of French Guiana (FG), a French overseas territory on the northern coast of South America.^2,3^ The same year, in FG, TONV was isolated from various genera of mosquitoes and sand flies: *Anopheles*, *Coquillettidia*, *Culex*, *Mansonia*, *Uranotaenia*, *Wyeomyia*, and *Lutzomyia*. In FG, between 1973 and 1977, TONV was isolated from 15 species of mosquitoes or sandflies but was most commonly detected in *Culex portesi*.^2,4^ The isolation of TONV from mosquitoes peaked at the beginning of the rainy season. In 1975, the virus was isolated from 14 sentinel newborn mice positioned in several locations surrounding the city of Cayenne.^2^ Surveys carried out between 1973 and 1977 to isolate arboviruses from wild mammals and birds yielded TONV isolates from 13 bird species but no isolates from the few collected species of wild mammals (*Chiroptera* sp., *Didelphis marsupialis*, and *Philander opossum*).^4^ Tonate virus was also isolated from mosquitoes in neighboring Suriname in a savannah area, principally in *C. portesi*.^5^ Tonate virus was also isolated from bugs (*Occiacus vicarius*) found in American cliff swallow nests (*Petrochelidon pyrrhonota*) in Colorado and Utah.^6^

There are a few publications about TONV and, to date, no human case has been described outside FG. Two human cases were reported in the 1970s, where TONV was isolated from blood, but a few clinical details were reported.^2^ The first patient was diagnosed in July 1973. He was living in Iracoubo, a small town in coastal FG, 150 km northwest of Cayenne. He presented with fever and headache and was coinfected with *Plasmodium falciparum.* The second case was reported in May 1975, in Cayenne, in a patient with mild symptoms: fever and headaches without arthralgia or myalgia.^2^ Two seroprevalence studies were conducted; one in the 1970s and the second in the 1990s, showing an overall anti-TONV IgG rate in the Guianese population of 14.3% and 11.9%, respectively.^2,7^ Seroprevalence rates did show significant spatial variation, ranging from 0% to 35% with the highest rates being reported from coastal areas in the 1970 survey, but in the 1990 survey, three communities located on the border rivers separating FG from Suriname and Brazil ranged from 42% to 53% compared with 19.5% in coastal areas. In the latter study, age-seroprevalence analysis showed an increase in seroprevalence with age, indicating endemic virus transmission.^7^ Nevertheless, the serological investigations could have detected antibodies to a closely related virus (at least in a subset of samples) instead of TONV. Comparative Dengue Plaque Reduction Neutralization Tests were not performed.

Concerning the clinical symptoms of TONV infection, few data are available. In the study published in 2001 by Talarmin et al.,^7^ 10 patients with TONV were reported: eight patients during the period between January 1997 and June 1999, in addition to the two already identified in 1975. These patients had a mild clinical presentation associated with fever (10/10), joint/muscle pains (4/10), headache (6/10), and digestive disorders (4/10). One fatal case was also described in 1998, in a 2-month-old infant who lived in Saint-Georges de l’Oyapock.^8^ He was hospitalized for encephalitis and died 72 hours after admission. The patient had elevated anti-TONV IgM in the blood, and the TONV genome was detected in a postmortem cerebral biopsy. Another serious case that could be attributed to TONV was published in 1997.^9^ A 3-year-old child was hospitalized for a febrile coma complicated by hepatic cytolysis and acute respiratory distress syndrome. A virus belonging to the VEE complex was isolated from the serum, but no cerebrospinal fluid virus isolation was performed for technical reasons (insufficient quantity). The clinical presentation for TONV infection is similar to that for other arbovirus infections, including dengue virus (DENV). Dengue virus is the most widespread arbovirus infection in the world, in general and in South America in particular.^10^ It is endemic and is responsible for periodic epidemics in FG, whereas for TONV, only sporadic cases have been reported. Tonate virus may thus be widely misdiagnosed notably when virological and serological tools are not available in routine.

The primary objective of this study was to describe the clinical presentation, including signs and symptoms and standard clinical testing for TONV infection. The secondary objective was to compare its characteristics with DENV infection to improve the clinical diagnosis.

## METHODS

### Setting.

French Guiana is a French overseas territory located on the northeastern coast of South America. About 90% of its 84,000 km^2^ surface is covered with the Amazonian rain forest; the remaining 10% in the north is covered by a coastal plain where 90% of the 269,352 inhabitants live. Cayenne and surroundings contain almost 50% of the population in 2016 (www.insee.fr). There are three main hospitals on the coast (Cayenne, Kourou, and Saint Laurent du Maroni), and there are 22 health centers in remote areas. As a part of France, FG has public health-care system, accessible for all legal and illegal residents.

### Study design and population.

An observational, retrospective, monocentric case–control study was conducted for the period between January 1, 2003, and August 31, 2016. Medical records were screened for patients presenting at the Centre Hospitalier Andrée Rosemon (CHAR) and patients from health centers in remote villages, for whom serum was sent to the National Reference Center for arboviruses (NRCA) at the Institut Pasteur de la Guyane (IPG) for arboviral serodiagnoses. All sera referred to the NRCA-IPG are tested for arboviruses circulating in FG, including DENV, TONV, Mayaro (MAYV), and chikungunya (Chikungunya virus [CHIKV], since 2014).

### Tonate virus cases.

Medical records for all patients testing positive for anti-TONV and/or anti-MAYV IgM were examined. Inclusion and exclusion criteria were then applied to each case as described in the following paragraphs.

### Dengue virus control cases.

To develop differential diagnosis criteria between TONV and DENV infections, we retrieved medical records from patients with positive DENV NS1 antigen identified at Cayenne Hospital laboratory during the 2013 DENV outbreak to use as gender- and age-matched controls. Tonate virus IgM–positive patients with DENV IgM were also excluded. Given the limited number of cases, to increase the significance of the study, we matched two DENV controls per TONV infection case. There were two gender- and age-matched DENV controls per case of positive TONV infection. For matching, we chose the control with the closest exact age to the age of the case.

### Exclusion and inclusion criteria and case definitions.

The exclusion criteria were the following: sera with cross-reactive antibodies with other alphaviruses (presence of IgM against other alphaviruses such as anti-CHIKV IgM associated with anti-MAYV or not), noninterpretable serology (such as positive IgM on day 0 or day 1 after the onset of the symptoms, decreasing titer between two successive samples), absence of available medical records, and, finally, certain alternative diagnosis. All patients without these exclusion criteria were re-evaluated by an adjudication committee composed of two infectious disease specialists from the CHAR and one virologist from the IPG. They classified patients into three categories.

1. High probability: typical clinical picture of arbovirus infection (i.e., with two or more of the following symptoms: fever, chills, headaches, myalgia, and arthralgia) and IgM seroconversion (appearance of IgM between two sequential samples collected 5 days apart)
2. Medium probability: typical clinical picture and single positive sample for IgM
3. Low probability: atypical clinical picture of infection and a single positive sample for IgM

To better describe TONV infection, only high and medium probability groups were retained in the analysis.

### Laboratory methods.

The detection of serum IgM antibodies to TONV was performed by the NRCA in FG using an in-house MAC-ELISA test modified from that previously described.^7^ Microtitration plates (Nunc Maxisorp; ThermoFisher Scientific, Roskilde, Denmark) were sensitized for 2 hours at 37°C with goat antihuman IgM (Sigma-Aldrich, St. Louis, MO) diluted in phosphate-buffered saline (PBS)–Tween buffer (PBS, 0.1% Tween 20). After three washes with PBS–Tween buffer, sera, positive and negative controls diluted (1/100) in PBS–Tween–milk buffer (PBS, 0.5% Tween 20, and 5% nonfat dry milk) were added to wells and incubated for 1 hour at 37°C. The wells were washed again and incubated overnight at 4°C in a humidified container with TONV or normal antigens diluted in PBS–Tween–milk buffer. Antigens were prepared by extracting TONV-infected or normal brains of suckling mice with sucrose–acetone. Specific antigen binding was demonstrated by using an ascitic fluid from TONV hyperimmune mice diluted in PBS–Tween–milk buffer incubated for 1 hour at 37°C, followed by incubation of a goat antimouse peroxidase–labeled conjugate (Sigma-Aldrich) diluted in PBS–Tween–milk buffer incubated for 1 hour at 37°C. Antigens and hyperimmune ascitic fluids are produced by the NRCA in FG. Three washes with PBS–Tween buffer were performed between each step. The 3,3,5,5′-tetramethylbenzidine liquid substrate system (Sigma-Aldrich) was used as the substrate. The titer of the optical density for TONV antigen in the patient serum divided by the optical density for negative TONV antigen in the same serum was measured. The presence of IgM against the studied virus was defined by a ratio higher than 3. Seroconversion was defined as the appearance of IgM between two sequential samples. A significant elevation was defined as a 2-fold or more increase of the ratios obtained during the same experiment on two successive samples taken at least 5 days apart.

### Data collection.

Clinical and biological data were collected by chart review using the Access^©^ software in August 2016, from the medical and biological software DMUnet^©^ (Dossier Médical des Urgences, CRIH des Alpes, France), CORA^©^ (Prismedica, Meylan, France), and SRI 3.083984 (Serveur de Résultats Intranet, AGFA HealthCare Enterprise Solutions^©^). The following variables were collected: age, gender, country of birth, occupation, town of residence, travel out of FG in the previous 15 days, medical history, date of symptoms onset, symptoms, usual laboratory features, and outcomes.

### Data analysis.

A simple descriptive analysis of clinical signs and symptoms and clinical laboratory results was carried out and presented with 95% CIs. Positive TONV cases were compared with DENV cases by a matched bivariate analysis using a conditional logistic regression model. The dependent variable was the diagnosis (TONV versus DENV). The explanatory variables included demographic, clinical, and biological features.

Continuous variables were transformed into categorical variables according to the standards of the Cayenne laboratory or according to the thresholds usually used in the literature. Age and temperature were transformed into binary variables: before 16 years of age (pediatric age), versus more than 15 years of age, and < 38°C versus ≥ 38°C. Biological variables were categorized according to laboratory thresholds as follows:

1. Hemoglobin as a binary variable: anemia if hemoglobin was less than 13 g/dL (male) or 12 g/dL (female)
2. Leukocytes in three classes: < 4,000/mm^3^ (leukopenia), 4,000–10,000/mm^3^, and > 10,000/mm^3^ (leukocytosis)
3. Neutrophils in three classes: < 1,500/mm^3^ (neutropenia), 1,500–7,500/mm^3^, and > 7,500/mm^3^ (neutrophilia)
4. Lymphocytes in three classes: < 1,500/mm^3^ (lymphopenia), 1,500–4,000/mm^3^, and > 4,000/mm^3^ (lymphocytosis)
5. Platelets in three classes: < 150,000/mm^3^ (thrombocytopenia), 150,000–450,000/mm^3^, and > 450,000/mm^3^ (thrombocytosis)
6. C-reactive protein (CRP) in two classes: ≤ 50 mg/L and> 50 mg/L; as classically used in the literature^11^
7. The presence of alanine aminotransferase and/or aspartate aminotransferase greater than two times the normal (80 U/L) was considered as cytolysis (binomial)
8. Glomerular filtration rate (binomial): < 60 mL/minutes (renal failure) and > 60 mL/minutes.^12^

The statistical analysis was carried out using R 3.2.3^©^ software.

### Ethics.

The retrospective analysis of medical records is authorized by the French regulatory agency Commission Nationale de l’Informatique et des Libertés (CNIL). All the data were confidential and collected on a standardized form, preventing any personal identification according to the procedures of the CNIL. The database was declared to the CNIL (n°2145898).

## RESULTS

### Characteristics of patients with TONV infection.

**Figure 1.**
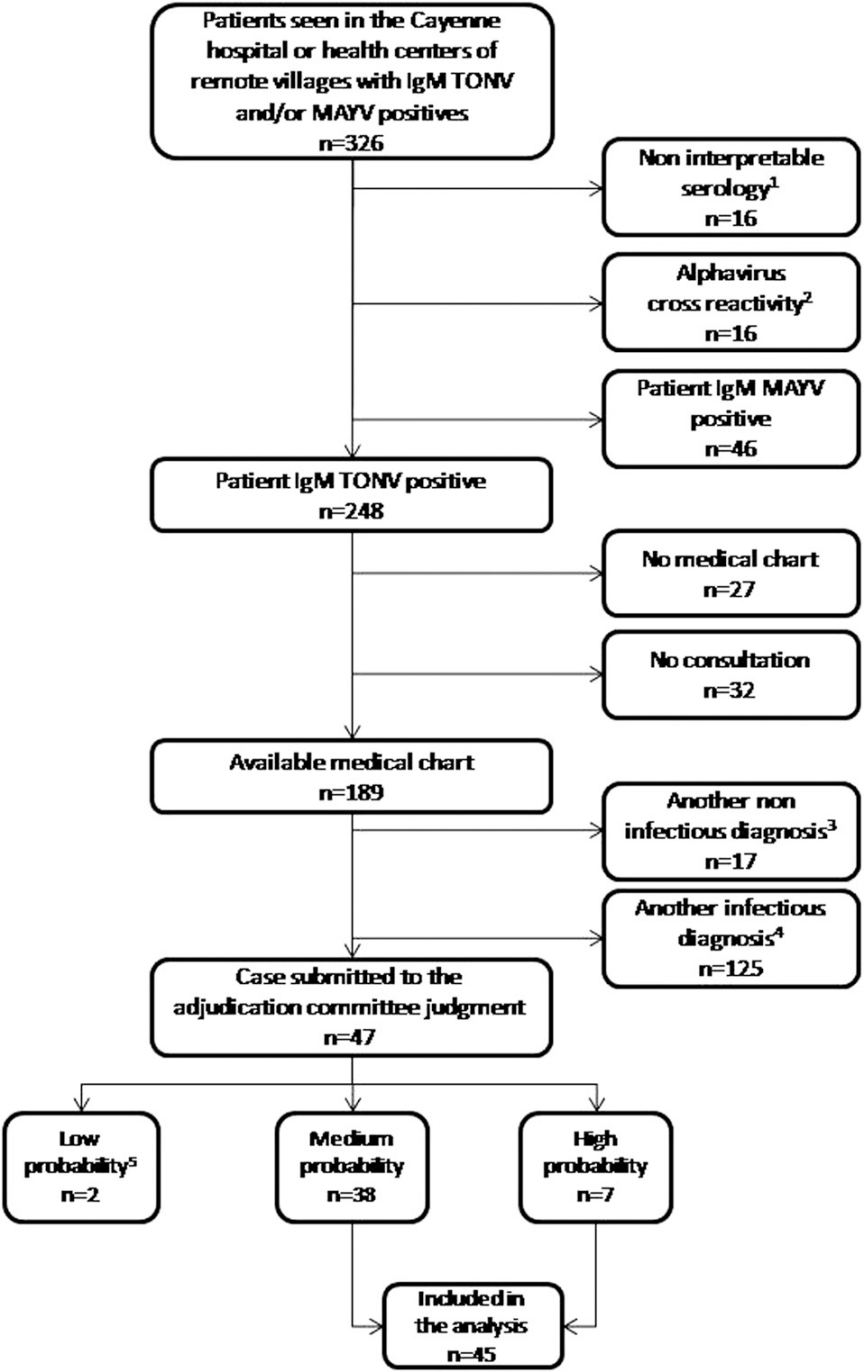
Flowchart of the study on Tonate virus infection in French Guiana, 2003–2016. MAYV = Mayaro Virus; TONV = Tonate Virus. ^1^Nine positive IgMs obtained 0–1 day after the onset of the symptoms; three with decreasing titer ratio between two successive samples, three with very slightly positive serology (negative ratio < 2, positive ratio > 3, and gray area between 2 and 3), and one with no information on the start date of clinical signs. ^2^Alphavirus cross-reactivity = reactivity to TONV and/or CHIKV and/or MAYV. ^3^Three deliveries, one pregnancy finding, one oral cancer, one atrial fibrillation, one renal colic, one myocardial infarction, one lung edema, one palpitation, two rheumatoid arthritis, one cirrhosis, one vaso-occlusive crisis, one atrial fibrillation, one pregnancy vomiting and one other. ^4^Thirty malaria attacks (19 *P. vivax*, nine *P. falciparum*, one coinfection, and two without species), 31 other arboviruses (17 chikungunya, 13 dengue, and one Zika), six angina (including three Epstein–Barr virus), 15 lung infections, five pyelonephritis, four leptospirosis, four Q fever, and 29 other infectious diagnoses. ^5^One persistent asthenia without fever and one infectious picture in a person with connective tissue disease with interstitial lung disease.

During the 13.5-year study period, 326 patients tested positive for IgM antibodies against TONV and/or MAYV. Two hundred forty eight patients had anti-TONV IgM antibodies, but no virus was isolated in culture. Of these, 59 were excluded because no medical data were available, 17 were excluded because of noninfectious alternative diagnoses, and 125 were excluded because of the presence of other infectious causes, including malaria and other arboviral infections (Figure 1). Forty-seven files were submitted to the adjudication committee: two were classified as low probability, 38 as medium probability, and seven as high probability. Forty-five patients with medium or high probability were included in the descriptive study of TONV infection. Among them, 17 (38%) were women (gender ratio M/F = 1.6), their median age was 34 years (IQR 25–75 = 22–49, range = 1–77), and 7/45 of them (16%) were 15 years old or less. Thirty-two (71.1%) patients had no underlying chronic disease. Of the remaining 13 patients, eight had cardiovascular risk factors (hypertension and dyslipidemia) and five had a history of chronic disease (lupus, tropical spastic paralysis, sickle cell anemia, hypothyroidism, and chronic alcoholism).

Tonate virus infection symptoms were characterized by self-reported fever (98%, 95% CI = 86–100%), headache (47%, 95% CI = 33–61%), myalgia (42%, 95% CI = 29–57%), and cough (33%, 95% CI = 21–48%) (Table 1), whereas clinical laboratory results were lymphopenia (43%, 95% CI = 28–58), anemia (33%, 95% CI = 20–48), and elevated transaminases (29%, 95% CI = 16–47) (Table 2). Five (11.1%) patients had an atypical presentation or a complication: one patient suffered from an acute meningoencephalitis, another from a meningeal syndrome, and three patients were labeled as acute hepatitis. No deaths were recorded.

**Table 1:** Clinical comparison of Tonate virus (TONV; *N* = 45) and dengue virus (DENV; *N* = 90) matched on gender and age in French Guiana, 2003–2016 (bivariate analysis)

Variables	TONV (*N* = 45)	DENV (*N* = 90)	OR (IC 95%)	*P* (Wald test)
	*n* (%)	*n* (%)		
Temperature > 38°C	18 (48.6)	54 (60.7)	0.5 (0.2; 1.2)	0.11
Fever	44 (97.8)	90 (100)	1	0.99
Chills	13 (28.9)	5 (11.4)	3.8 (0.95; 15)	0.06
Headache	**24** (**53.3)**	**63** (**70.8)**	**0.3** (**0.1; 0.8)**	**0.02**
Joint pain	12 (26.7)	13 (15.7)	1.7 (0.7; 4.1)	0.2
Muscle pain	**19** (**42.2)**	**54** (**61.4)**	**0.4** (**0.2; 0.9)**	**0.02**
Low back pain	12 (26.7)	21 (25)	1.1 (0.5; 2.4)	0.8
Retro-orbital pain	8 (17.8)	24 (29.3)	0.5 (0.2; 1.3)	0.1
Fatigue	**8** (**17.8)**	**34** (**41)**	**0.3** (**0.1; 0.7)**	**0.01**
Rash	**1** (**2.2)**	**23** (**27.4)**	**0.06** (**0.01; 0.4)**	**0.01**
Pruritus	1 (2.2)	8 (9.1)	0.2 (0.02; 1.6)	0.1
Confusion	1 (2.2)	3 (3.7)	0.6 (0.05; 7.4)	0.7
Abdominal pain	8 (17.8)	20 (22.7)	0.7 (0.3; 1.8)	0.4
Anorexia	**3** (**6.7)**	**25** (**30.1)**	**0.2** (**0.06; 0.6)**	**0.01**
Nausea	**2** (**4.4)**	**28** (**32.2)**	**0.1** (**0.02; 0.5)**	**< 0.01**
Vomiting	9 (20)	20 (22.7)	0.8 (0.3; 2.1)	0.7
Diarrhea	3 (6.7)	16 (18.2)	0.4 (0.1; 1.3)	0.1
Cough	**15** (**33.3)**	**9** (**10.3)**	**5.6** (**1.8; 17.1)**	< 0.01

Bold values indicate variables with significative difference (*P* < 0.05).

**Table 2:** Main biological variables of patients with probable Tonate virus infection (*N* = 45) in French Guiana, 2003–2016

Variables	Mean	Median	First quartile	Third quartile	ET
Hemoglobin (g/dL)	13.13	13.10	12.45	14.22	2
Leukocytes (/mm^3^)	8,133	6,900	4,900	9,800	4,533
Polynuclear neutrophil (/mm^3^)	4,548	4,100	2,290	5,560	3,569
Lymphocytes (/mm^3^)	2,172	1,662	1,041	2,336	2,131
Platelet (/mm^3^)	259,146	238,000	148,000	313,000	165,254
C-reactive protein (mg/dL)	23.6	11.8	3.6	23.5	34.8
Aspartate aminotransferase	11.6	11	6.5	16.5	6.6
Alanine aminotransferase	12.5	12	6.25	18	7.5

### Tonate virus acute meningoencephalitis case report.

The patient with acute meningoencephalitis was a 29-year-old woman born in FG who initially presented fever, chills, shoulder pain, and headache for 10 days. Then, her condition worsened with agitation, spatiotemporal disorientation, intense headache, photophobia, and neck stiffness. The Kernig and Brudzinski signs were positive. Brain computerized tomography scan and magnetic resonance imaging were normal. Blood biological tests revealed CRP elevation (165 mg/L) without leukocytosis (4,100/mm^3^) or thrombocytopenia (198 G/L). Cerebrospinal fluid (CSF) samples revealed the following features: the white blood cell count was 400/mm^3^ (70% neutrophils and 30% lymphocytes), blood cell count was 2/mm,^3^ protein concentration was 1.52 g/L, and glucose concentration was 3.20 mmol/L (concomitant blood glucose was 5.6 mmol/L). Regarding the search for etiology, only anti-TONV IgM was positive in CSF, and TONV polymerase chain reaction (PCR) could not be performed. Direct examination and culture for acid/alcohol–fast bacilli, *Streptococcus pneumoniae* soluble antigen, cytomegalovirus, human herpes virus 6 (HHV6), herpes simplex virus, varicella zoster virus (VZV), Epstein–Barr virus (EBV), *Mycoplasma pneumoniae*, and *Toxoplasma gondii* PCR were negative in CSF. All other infectious investigations were negative (thick and thin blood smear for malaria, cultures on blood, urine and stool, chikungunya PCR, serodiagnosis for *M. pneumoniae*, hepatitis B and C virus, human immunodeficiency virus, HHV6, VZV, EBV, toxoplasmosis, chikungunya, dengue, and MAYV).

The clinical evolution was favorable in 3 days without any neurological sequelae. Cerebrospinal fluid abnormalities disappeared on the lumbar puncture performed 6 days after the first one.

### Comparison between TONV and DENV clinical and biological characteristics.

Bivariate analysis suggested that TONV infections are not associated with headache, myalgia, asthenia, cutaneous rash, anorexia, and nausea when compared with DENV cases. Tonate virus infections were more often associated with the presence of cough than DENV cases. The laboratory indicators associated with TONV versus DENV were the presence of anemia, the absence of leukopenia, and the lymphocyte count less than 1.5 G/L (Table 3).

**Table 3:** Biological comparison of Tonate virus (TONV; *N* = 45) and dengue virus (DENV; *N* = 90) matched on gender and age in French Guiana, 2003–2016 (bivariate analysis)

Variables		TONV (*N* = 45)	DENV (*N* = 90)	OR (IC 95%)	*P* (Wald test)
		*n* (%)	*n* (%)		
Anemia	woman < 12 g/dL; man < 13 g/dL	13 (32.5)	10 (11.1)	6.6 (1.8; 23.8)	< 0.01
Leukocytes	< 4,000/mm^3^	4 (9.8)	40 (44.4)	0.09 (0.02; 0.4)	< 0.01
Neutrophils	< 1,500/mm^3^	6 (14.6)	18 (20.2)	0.61 (0.2; 1.7)	0.3
Lymphocytes	**< 1,500/mm**^**3**^	**17** (**42.5)**	**80** (**89.9)**	**0.09** (**0.03; 0.3)**	**< 0.01**
Platelets	< 150,000/mm^3^	11 (26.8)	48 (53.3)	0.4 (0.01; 1.03)	0.056
C-reactive protein	> 50	8 (20)	7 (8.2)	2.6 (0.9; 7.4)	0.08
Transaminase elevation	Aspartate aminotransferase and/or alanine aminotransferase > 2N	9 (29)	14 (17.1)	1.8 (0.6; 5.4)	0.3
Glomerular filtration rate	< 60 mL/minutes	7 (8.5)	2 (6.7)	0.6 (0.1; 3.4)	0.6
Hospitalization	–	10 (22.2)	22 (24.4)	0.9 (0.4; 2.1)	0.8

Glomerular filtration rate according to the MDRD (Modification of the Diet in Renal Disease) formula. Bold values indicate variables with significative difference (*P* < 0.05).

## DISCUSSION

We here present the largest epidemiological and clinical study to date on TONV infection. The previous clinical description only reported 10 cases.^7^ The number of patients with a diagnosis of TONV infections has been increasing lately because of a more systematic screening of arbovirus-like clinical presentations. In addition, the 2013–2014 chikungunya outbreak resulted in a large increase in blood samples sent to the NRCA in Cayenne, and subsequently to more searches for TONV infection. Nevertheless, the results of this study should be interpreted keeping several possible biases in mind. The retrospective data collection could be responsible for misdiagnosis and misclassification of TONV and DENV infections. This is why we excluded any TONV cases if there was any doubt of an alternative diagnosis or coinfection. Conversely, DENV-infected patients were only selected using AgNS1 positivity, without eliminating any coinfections.

There is an impressive contrast between the frequent diagnosis of TONV in FG and its “lack of detection” in the surrounding countries because of FG’s special position. Indeed, it is an overseas French territory, with European human, technical, and financial resources available for medicine and research in the northeastern region of the Amazon. Thus, FG is a “sentinel territory” for the other Amazonian countries (Suriname, Guyana, Bolivar state in Eastern Venezuela and Amapá state in northern Brazil), with the means to detect rare or less known pathogens. Thus, other infectious agents are routinely diagnosed in FG, whereas they seem to be absent or rare in the surrounding countries such as Q fever or disseminated histoplasmosis in AIDS patients.^13,14^ Tonate virus is probably present in neighboring countries because borders have never stopped the circulation of viruses, but it is not diagnosed because of its nonspecific presentation leading to confusion with infections with DENV, CHIKV, or Zika virus. In addition, the serodiagnosis of this virus has not been implemented in the surrounding countries, and it is not searched in case of arbovirus-like infection when the more classical diagnoses have been ruled out.

Although the symptoms associated with TONV infection appears to be essentially mild, particular attention must be given to the possibility of neurological forms, as highlighted by the meningoencephalitis case report in this study, in addition to the two cases previously reported in FG.

The neurological tropism is not surprising because TONV belongs to the VEE complex. It was already described as neurovirulent in mice and Guinea pigs after intracerebral inoculation, but not after intraperitoneal inoculation.^3^ For subtypes IAB, IC, and ID, 0.5–4% of neurological forms are described in humans. Neurological cases have been described for the subtypes Everglades (II) and Mena (IE).^15,16^ In conclusion, other alphaviruses are also responsible for neurological diseases in another antigenic complex of Alphavirus, such as Eastern and Western equine encephalitis and chikungunya viruses.^17,18^

The comparison between patients with TONV and DENV shows that TONV infection corresponds to “dengue-like syndrome” with a milder presentation. Tonate virus and DENV infections can easily be confused. In our study, only cough and anemia were significantly associated with TONV. These signs are not sensitive enough to clinically distinguish the two infections. The same applies to other EEV viruses.^19^ In a study conducted in Ecuador, Peru, Bolivia, and Paraguay on febrile syndromes, 2.1% were due to VEE virus under type ID, whereas 26% were due to DENV.^20^ Thus, the presence of the “eyes nose throat” signs in 11% of patients with TONV infection probably leads to confusion between the usual pathogens of the ear, nose and throat sphere and this arbovirus.

The possibility of neurological complications associated with a nonspecific bio-clinical picture confirms that it is reasonable to look for TONV infection in febrile syndromes in patients living or returning from countries of the Guiana Shield. However, in our series, 133/208 (63.9%) patients had positive serodiagnoses in favor of coinfection with other pathogens, and 16/280 (5.7%) had cross-reaction serodiagnoses between alphaviruses. Therefore, the presence of a single positive TONV IgM should not stop further diagnostic investigations, leading to misdiagnosis and possible detrimental patient outcomes.

However, even if practitioners think about TONV infection, its microbiological diagnosis is not easy. Isolation of viruses of the VEE complex is difficult because viremia is generally short (3–5 days as many arboviruses), and low and TONV viremia has never been described yet.^21,22^ Cooperation between clinicians and the laboratory must be improved to facilitate cultures of blood samples taken in the first days of illness. The more systematic collection of blood samples would make it possible to identify viruses that were not specifically suspected. On the other hand, the isolation of new strains of TONV would allow the sequencing of these arboviruses, and the increase in available sequences would allow improving of our knowledge of the genetic diversity of these viruses. This could improve the intrinsic characteristics of the PCR methods, which are very important to improve the identification of such infections in a context where accurate serologic methods are lacking. Indeed, in FG with endemic cocirculation of several alphaviruses, potential cross-reactive antibody responses make the differentiation among alphavirus infections and the confirmation of a specific virus infection difficult. Even neutralization assays, usually used to measure virus-specific neutralizing antibodies, could be affected by the production of cross-reactive antibodies early after infection and/or after several alphavirus infections as already largely described for flaviviruses.^23,24^

In the past 10 years, two arboviruses which were almost unknown have emerged and circulated worldwide. Chikungunya emerged in 2006 in la Reunion Island (Indian Ocean) and then spread worldwide, responsible for acute and chronic arthritis.^25^ Zika virus emerged in 2007 in Yap Island (Pacific Ocean) then spread to French Polynesia^26^ and South America, responsible for neurological complications (Guillain–Barré syndrome and fetal microcephaly).^27,28^ Thus, we should pay attention to any arbovirus that is pathogenic for humans because it has the potential to spread beyond its initial area of endemicity. Several animals, particularly rodents, are part of the endemic cycle of EEV viruses,^19,29^ but migratory birds may be able to spread TONV in many American countries.^6^ The migratory birds that pass through FG follow well-known migration routes: the eastern coast of Canada and the United States of America, the Gulf of Mexico and the Caribbean, the Atlantic coast of Brazil, and Argentina.^30^ Indeed, TONV has already been found in the bugs of American cliff swallow nests.^6^

## CONCLUSION

The clinical and biological presentation of TONV infection, which has never been described in humans outside the French Guianese borders, is unspecific and difficult to differentiate from dengue fever, but benign most of the time. Neurological presentations are, however, possible and can be fatal. Thus, the infection should be considered in patients with fever and/or febrile neurological signs living or returning from the area. Tonate virus should be in the differential diagnosis for a host of arboviral infections that are endemic in FG and other South American countries. Further studies are needed to develop easier tools, such as PCR for the routine diagnosis of this arboviral infection and scale them up to surrounding countries where the infection probably exists. Nevertheless, in endemic areas, a positive serodiagnosis for TONV may not rule out other diagnoses because there is a high cross-reactivity with other alphaviruses, but it may also be positive during infections by other viruses, bacteria, or parasites.
